# Highly Photostable and Luminescent Donor–Acceptor Molecules for Ultrasensitive Detection of Sulfur Mustard

**DOI:** 10.1002/advs.202002615

**Published:** 2021-01-04

**Authors:** Linfeng Cui, Yanjun Gong, Chuanqin Cheng, Yongxian Guo, Wei Xiong, Hongwei Ji, Lang Jiang, Jincai Zhao, Yanke Che

**Affiliations:** ^1^ Beijing National Laboratory for Molecular Sciences Key Laboratory of Photochemistry Institute of Chemistry Chinese Academy of Sciences University of Chinese Academy of Sciences Beijing 100049 China; ^2^ Key Laboratory of Colloid and Interface Chemistry Ministry of Education School of Chemistry and Chemical Engineering Shandong University Jinan 250100 China

**Keywords:** donor–acceptor molecules, fluorescence sensors, high emission efficiency, photostability, sulfur mustard

## Abstract

Real‐time, high signal intensity, and prolonged detection is challenging because of the rarity of fluorophores with both high photostability and luminescence efficiency. In this work, new donor–acceptor (D–A) molecules for overcoming these limitations are reported. A hybridized local and an intramolecular charge‐transfer excited state is demonstrated to afford high photoluminescence efficiency of these D–A molecules in solution (≈100%). The twisted molecular structure and bulky alkyl chains effectively suppress *π*–*π* and dipole–dipole interactions, enabling high luminescence efficiency of **1** and **2** in the solid state (≈94% and 100%). Furthermore, two D–A aggregates exhibit high photostability as evidenced by 4% and 8% of the fluorescence decreasing after 6 h of continuous irradiation in air, which is in sharp contrast to ≈95% of fluorescence decreasing in a reference compound. Importantly, with these molecules, ultrasensitive detection of sulfur mustard (SM) with a record limit of 10 ppb and selective detection of SM in complex matrices are achieved.

## Graphical abstract

Two new donor–acceptor molecules that exhibit high photostability and photoluminescence efficiency (close to unity) both in solution and in the solid state are developed. Their application in fluorescence sensor is well illustrated by utilizing **1** porous films to ultrasensitively and selectively detect 10 ppb sulfur mustard vapor.

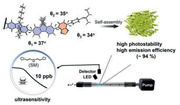

For the application of organic fluorophores, high photostability and strong luminescence are significantly important parameters.^[^ ^1^, ^2^, ^3^, ^4^ ^]^ In particular, under high (de)excitation light intensity (e.g., stimulated emission depletion microscopy) and long‐term light exposure (e.g., real‐time fluorescence sensors), fluorophores with high photostability and luminescence efficiency enable the improvement of imaging resolution and a prolonged observation window.^[^ ^2^, ^3^, ^4^ ^]^ In contrast, fluorophores with low photostability and weak luminescence cause the loss of imaging resolution and signal intensity as well as a reduced working life. While substantial progress has been made in several organic systems to improve fluorophore brightness and photostability,^[^ ^1^, ^2^, ^3^, ^4^, ^5^, ^6^, ^7^, ^8^, ^9^ ^]^ it remains a great challenge to develop fluorophores that exhibit high photostability and emission efficiency in an aggregated state.

As a widely explored class of fluorophores, organic donor–acceptor (D–A) molecules have the potential for the construction of fluorophores with high photostability and luminescence efficiency.^[^ ^10^, ^11^, ^12^, ^13^, ^14^, ^15^, ^16^, ^17^, ^18^, ^19^, ^20^, ^21^, ^22^ ^]^ First, introduction of the appropriate A moiety can effectively lower the highest occupied molecular orbital (HOMO) and lowest unoccupied molecular orbital (LUMO) of the resulting D–A molecules, which can enhance their photostability.^[^ ^10^, ^12^, ^13^, ^14^ ^]^ Second, the twisting geometry of D–A molecules allows for a certain extent of orbital mixing, producing a hybridized local excited‐state (LE) and intramolecular charge‐transfer (CT) state (i.e., a hybridized local and an intramolecular charge‐transfer (HLCT) excited state).^[^ ^19^, ^20^, ^21^, ^22^ ^]^ The resulting HLCT state can provide a high‐efficiency luminescent pathway for D–A molecules. Furthermore, optimal D and/or A moieties offer molecular recognition of a specific analyte, which, along with high photostability and luminescence efficiency, makes D–A molecules suitable for constructing fluorescence sensors. Despite the promising characteristics described above, few D–A molecules haven been reported to show high photostability and luminescence efficiency of >70% both in solution and in the solid state.^[^ ^4^, ^11^, ^21^ ^]^ This is mainly because *π*‐*π* and dipole‐dipole interactions of D–A molecules can greatly reduce their luminescence in the solid state,^[^ ^23^ ^]^ even though they are highly luminescent in solution. Therefore, rational design of D–A molecules with high photostability and luminescence efficiency both in solution and in the solid state is highly desirable.

In the present work, we design new D–A molecules **1** and **2** (**Figure** **1**) that exhibit high photostability and luminescence efficiency (>90%) both in solution and in the solid state. We demonstrate that the HLCT state formed in twisted **1** and **2** allows for a luminescence efficiency of up to unity in solution. Furthermore, the twisted D–A structure and bulky alkyl chains suppress *π*‐*π* interactions in the solid state and thereby generate a high luminescence efficiency (close to unity) of solid films of molecules **1** and **2**. On the other hand, the benzothiadiazole groups (A moieties) effectively lower the HOMO and LUMO of molecules **1** and **2**, thus offering greatly enhanced photostability. The high luminescence efficiency and photostability make these molecules suitable for fluorescence sensors. In particular, porous **1** films that have optimized sulfur‐*π* and dipole‐dipole interactions with sulfur mustard (SM) enable the ultrasensitive detection of 10 ppb SM. Furthermore, porous **1** films exhibit no responses to most potential interferents, which thereby allow for the selective detection of SM in complex environments. These results provide important insights into the synthesis and application of organic fluorophores with high photostability, as well as, high luminescent efficiency.

**Figure 1:**
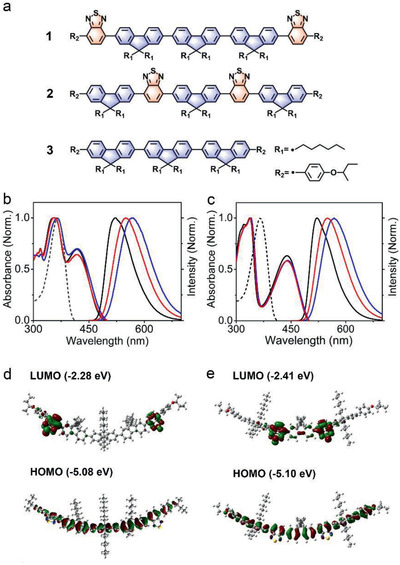
a) Molecular structures of **1**–**3**. Normalized UV–vis absorption spectra and fluorescence spectra of b) **1** and c) **2** in cyclohexane (black), chloroform (red), and *N*,*N*‐dimethylformamide (blue) solution (2.5 µM). Note that the dashed lines are normalized UV–vis absorption spectra of free **3** in dichloromethane. DFT‐calculated frontier molecular orbitals of molecules d) **1** and e) **2**.

The detailed synthesis and characterization of **1–2** (Figure 1a) are provided in the Supporting Information. For comparison, molecule **3** was also synthesized by following a previously reported method.^[^ ^24^ ^]^ As shown in Figure 1a, molecules **1** and **2** have the same D (fluorene) and A (benzothiadiazole) moieties but are different in their linking sequence, while molecule **3** has only the same D moiety. Here, the designed molecules **1** and **2** that have bulky side chains and different electronic moieties were expected to exhibit a twisted molecular structure. As shown in Figure S1, Supporting Information, density‐functional theory (DFT) calculations confirm that the twisted angles between the D or A moieties in molecules **1** and **2** fall within the range of 30°–35°. Similar to other D–A molecules,^[^ ^20^, ^21^, ^22^, ^23^, ^25^, ^26^, ^27^, ^28^, ^29^, ^30^ ^]^ the twisted molecular structure of **1** and **2** may allow for the formation of an intercrossed LE and CT state that can provide a pathway for high emission efficiency. To support this hypothesis, we performed optical measurements of **1** and **2** in solvents with different polarities. As shown in Figure 1b, the absorption peak of **1** centered at 411–420 nm, compared to that of **3** centered at ≈366 nm, can be ascribed to the CT state of **1**. The CT characteristic is further confirmed by the obvious redshift of the fluorescence spectra with the increasing solvent polarity. For example, the fluorescence peak of **1** redshift from 522 nm in cyclohexane to 567 nm in dimethylformamide. Similarly, molecule **2** exhibits a CT character of the excited state, as evidenced by the emergent absorption peak centered at 436–441 nm and the obvious redshift of the fluorescence spectra with increasing solvent polarity (Figure 1c). The obvious redshift of the fluorescence spectra with the increasing solvent polarity should result from the strong interaction between the solvent field and the CT excited state that has a large dipole moment as confirmed by theoretical calculations below. Interestingly, both **1** and **2** have the fluorescence quantum yields (*Φ*) of ≈100% in the different solvents (Table S1, Supporting Information), which is an uncommon phenomenon for the pure CT excited state.^[^ ^31^, ^32^, ^33^ ^]^ We measured the extinction coefficient (*ε*) of **1** and **2** in chloroform solution to be 82 640 and 77 000 L mol^−1^ cm^−1^ (Figure S2, Supporting Information), which thereby gave rise to the brightness (*ε* × *Φ*) of 82 640 and 77 000 for **1** and **2** in chloroform, respectively.

To further explore the low‐lying excited state (S_1_) properties, we used the Lippert‐Mataga equation^[^ ^34^ ^]^ to fit the Stokes’ shift and the orientation polarizability (Table S1, Supporting Information). As shown in Figure S3, Supporting Information, the linear relation for **1** and **2** is different in low‐polarity and high‐polarity solvents. The corresponding excited‐state dipole moment (*µ*_e_) for **1** and **2** is calculated to be 17.6 D and 17.2 D in low‐polarity solvents and 24.4 D and 23.7 D in high‐polarity solvents, respectively. The small *µ*_e_ of 17.6 D and 17.2 D can be attributed to the usual LE‐like state of **1** and **2**, while the large *µ*_e_ of 24.4 D and 23.7 D can be attributed to the CT‐like state of **1** and **2**. The nonlinear relationship between Stokes’ shift and solvent polarity (Figure S3, Supporting Information) indicates that **1** and **2** possess an intercrossed excited state of LE and CT, that is, HLCT, in moderate polarity solvents.^[^ ^21^, ^22^ ^]^ Other experimental evidence for the formation of the HLCT state was observed from fluorescence lifetime measurements of **1** and **2** in a moderately polar solvent. The fluorescence of **1** and **2** in chloroform follows single‐exponential decay (Figure S4, Supporting Information), giving lifetimes of 6.41 and 4.54 ns, respectively; these results indicate that **1** and **2** have an HLCT state rather than two separate states.^[^ ^20^ ^]^ The results are also consistent with the frontier orbital data calculated by Gaussian 09 package. As shown in Figure 1d,e, the HOMO of **1** and **2** is distributed on the whole molecular backbone, whereas the LUMO is mainly located on the benzothiadiazole unit with some extent of distribution on the fluorene unit. The insufficient separation of the HOMO and LUMO levels likely leads to the intercrossed characteristics of the LE and CT states. Importantly, the formed HLCT state could provide access to a radiative process and thus gives rise to the high emission efficiency of **1** and **2** (Table S1, Supporting Information).

We next explored the solid‐state emission of **1** and **2**. Aggregates from **1** or **2** were fabricated by simply injecting 0.3 mL of **1** or **2** in a chloroform solution (6 mg mL^−1^) into a vial containing 3 mL of methanol followed by 1 day of aging. Scanning electron microscopy (SEM) revealed that molecules **1** and **2** formed irregular colloidal nanoparticles (NPs）‐like structures with lengths of hundreds of nanometers (**Figure** **2**a,b). When excited by 350–400 nm UV light, these colloidal NPs are highly emissive (Figure 2c,d). The fluorescence quantum yield (FQY) values of colloidal NPs from **1** and **2** were measured to be ≈94% and 100%, respectively, using a calibrated integrating sphere system. We considered that the twisted molecular structure and bulky alkyl chains in **1** and **2** could suppress *π*‐*π* and dipole‐dipole interactions in the solid state to allow for a high luminescence efficiency. To support this, we compared the optical spectra of colloidal NPs from **1** and **2** with those of individual molecules in solution. As shown in Figure 2e,f, the absorption and fluorescence spectra of these colloidal NPs remain almost the same as those of the corresponding molecules in chloroform solution, indicative of the negligible *π*‐*π* and dipole‐dipole interactions in the resulting colloidal NPs. X‐ray diffraction measurements of colloidal NPs from **1** and **2** showed no diffraction peaks (Figure S5, Supporting Information), further indicating that the twisted structure and bulky alkyl chains resulted in the amorphous structure of colloidal NPs from **1** and **2**.

**Figure 2:**
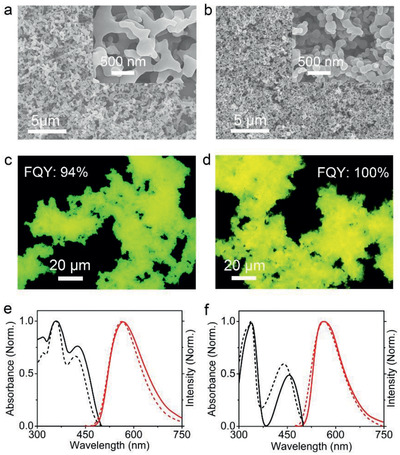
SEM images of a) colloidal NPs from **1** and b) colloidal NPs from **2**. Inset: Magnified SEM image of colloidal NPs from **1** and **2**. Fluorescence‐mode optical microscopic image of c) colloidal NPs from **1** and d) colloidal NPs from **2**. e) Normalized absorption (black) and fluorescence spectra (red) of colloidal NPs from **1** (solid) cast on a quartz slide and **1** monomers (dashed) in dichloromethane (2.5 µM). f) Normalized absorption (black) and fluorescence spectra (red) of colloidal NPs from **2** (solid) cast on a quartz slide and **2** monomers (dashed) in dichloromethane (2.5 µM).

For practical applications, photostability is another important parameter.^[^ ^35^ ^]^ The photostability of colloidal NPs from **1** and **2** was thus evaluated by monitoring the fluorescence intensity as a function of the continuous irradiation time. As shown in **Figure** **3**a,c, the fluorescence intensity of colloidal NPs from **1** decreases by only 4% after 6 h of continuous irradiation in air (385 nm, 0.053 mW cm^−2^), and the fluorescence intensity of colloidal NPs from **2** decreases by ≈8% under identical irradiation conditions. In sharp contrast, the fluorescence intensity of aggregates from **3** decreases by ≈95% under identical irradiation conditions (Figure S6, Supporting Information). Notably, even under irradiation in air by a 365 nm LED lamp (100 mW cm^−2^), the fluorescence intensity of colloidal NPs from **1** and **2** decreases by only ≈8% and ≈10%, respectively, after 6 h of continuous irradiation (Figure S7, Supporting Information). These results indicate the high photostability of colloidal NPs from **1** and **2**. Such high photostability is also reflected by the time‐dependent fluorescence spectra of these colloidal NPs, where the fluorescence spectra only slightly decreases under continuous UV irradiation (Figure 3b,d and Figure S7, Supporting Information). To explain the origin of high photostability, we used DFT to calculate and analyze the energy levels of the HOMO and LUMO of **1** and **2** (Figure 1d,e). Compared to molecule **3** (Figure S8, Supporting Information), the introduction of benzothiadiazole moieties in molecules **1** and **2** largely lowers the LUMO energy level by approximately 1 eV. The lowered LUMO energy level can greatly reduce the possibility of losing an electron from the LUMO to oxygen; this thereby can greatly enhance the stability of **1** and **2** in the excited state. In addition, inefficient intersystem crossing from the singlet state to the triplet state in **1** and **2**, consistent with their high FQYs described above, can also play an important role in enhancing photostability.^[^ ^1^ ^]^

**Figure 3:**
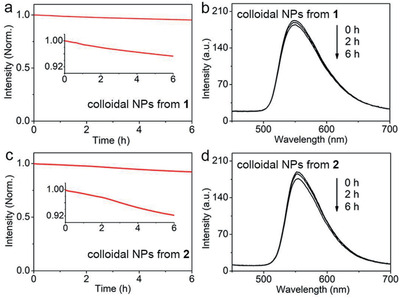
Fluorescence intensity of colloidal NPs from a) **1** and c) **2** monitored in the range of 520–560 nm as a function of irradiation time (385 nm, 0.053 mW cm^−2^). The insets shows zoomed‐in plots. Time‐dependent fluorescence spectra of colloidal NPs from b) **1** and d) **2** as a function of irradiation time (385 nm, 0.053 mW cm^−2^).

The high photostability and luminescence efficiency of **1** and **2**, which can also form porous colloidal NPs once deposited on substrates, enable them to be suitable fluorescence sensors for vapor analytes. Here, we chose a powerful blistering agent, SM, as the target analyte to illustrate the sensing application of these D–A molecules. SM belongs to a typical class of chemical warfare agents.^[^ ^36^, ^37^, ^38^ ^]^ Furthermore, there is no treatment or antidote for injuries caused by SM gas. To date, examples of fluorescent detection of trace SM vapor remain very limited.^[^ ^24^, ^39^, ^40^, ^41^, ^42^, ^43^ ^]^ Therefore, ultrasensitive detection of SM vapor by a reliable fluorescence sensor is critical. Quantitative evaluation of the detection sensitivity was performed at the Chemical Defense National Laboratory, where SM vapors at different concentrations were available. The sensing experiments of colloidal NPs from **1** and **2** were performed using a home‐built optical chamber coupled with an Ocean Optics USB4000 fluorometer.^[^ ^44^, ^45^ ^]^

As shown in **Figure** **4**a,c, exposure of colloidal NPs from **1** and **2** to trace SM vapors leads to obvious irreversible fluorescence quenching responses. Such fluorescence quenching was also reflected clearly in the fluorescence spectral changes (Figure 4b,d). Notably, colloidal NPs from **1** enabled the detection of SM at a concentration as low as 10 ppb. In contrast, colloidal NPs from **2** exhibited less sensitivity, where SM vapor was detected at 60 ppb under identical conditions. Compared to colloidal NPs from **2**, colloidal NPs from **1** likely have enhanced noncovalent interactions with SM and thereby increased the detection sensitivity. To support this, we performed theoretical calculations to compare noncovalent interactions between molecules **1–2** and SM. As shown in Figure S9, Supporting Information, the binding energy between **1** and SM is calculated to be 19.8 kcal mol^−1^, which is larger than that between **2** and SM (18.1 kcal mol^−1^). The noncovalent interactions mainly originate from sulfur−*π* interactions and dipole‐dipole interactions between the D–A molecule and SM. Therefore, the stronger interactions of **1** with SM than of **2** with SM enhanced the detection sensitivity of colloidal NPs from **1**.

**Figure 4:**
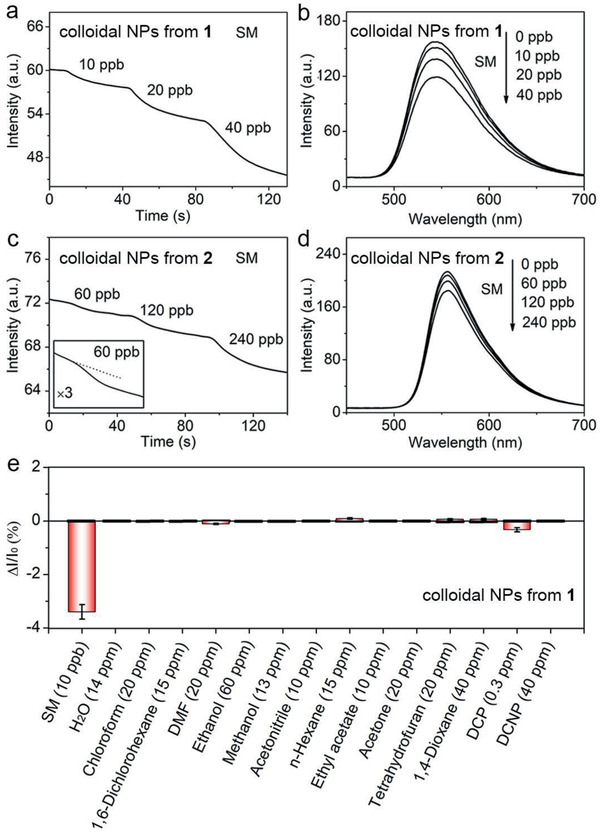
Time‐course curves of the fluorescence responses of a) colloidal NPs from **1** and c) colloidal NPs from **2** monitored in the range of 520–560 nm upon exposure to SM vapors at different concentrations. Fluorescence spectra of b) colloidal NPs from **1** and d) colloidal NPs from **2** when exposed to SM vapors at different concentrations. e) Fluorescence responses of colloidal NPs from **1** to SM and various potential interferences. Error bars represent the standard deviation of five measurements.

Importantly, colloidal NPs from **1** exhibited no responses to a nerve agent simulant, diethyl cyanophosphonate, and most common organic solvents (Figure 4e and Figures S10 and S11a, Supporting Information), likely because they did not undergo strong interactions with **1** to induce signal responses. Another nerve agent simulant, diethyl chlorophosphate, at a relatively high concentration can cause moderate fluorescence quenching (Figure S11b, Supporting Information). However, the quenching is reversible, which is distinct from the irreversible responses quenching by SM vapor and thereby does not interfere with SM detection (Figure 4a). Notably, different sulfides can cause irreversible fluorescence quenching (Figure S12, Supporting Information) because of sulfur‐*π* interactions.^[^ ^46^, ^47^, ^48^ ^]^ To discriminate SM from various sulfides, although rarely encountered, we could fabricate a two‐member sensor array consisting of colloidal NPs from **1** and aggregates from **3**. As illustrated in Figure S13, Supporting Information, the distinct combined responses induced by differentiated noncovalent interactions^[^ ^24^ ^]^ allowed for the simple discrimination of SM from common sulfides.

Finally, the detection of SM in complex environments was carried out to illustrate the utility of colloidal NPs from **1** in a real‐world sensing application. Car exhaust gas containing complex emission components, and hair spray containing ethanol, butane, isobutane, panthenol, flavors, etc., were employed as complex matrices that may potentially interfere in SM analysis. As shown in **Figure** **5**a, exposure of colloidal NPs from **1** to the car exhaust gas slightly enhanced the fluorescence responses, which quickly recovered. In contrast, the addition of trace SM in the car exhaust gas caused the irreversible quenching of colloidal NPs from **1** right after the fluorescence increase by the car exhaust gas matrix (Figure 5b). These results indicate that SM vapor, even at 20 ppb in car exhaust gas, can be distinguished by colloidal NPs from **1**. When exposed to the hair spray, colloidal NPs from **1** exhibited no fluorescence responses (Figure 5c), consistent with the above observations that colloidal NPs from **1** were insensitive to most organic solvents (Figure S10, Supporting Information). Upon exposure to the mixture of trace SM and hair spray, irreversible fluorescence quenching responses of colloidal NPs from **1** were clearly observed (Figure 5d). These results demonstrate the capability of colloidal NPs from **1** in detecting trace SM in the presence of complex interferents and the potential for their commercialization.

**Figure 5:**
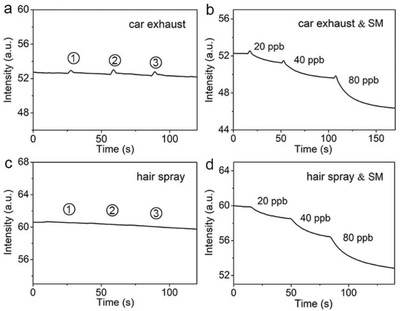
Time‐course curves of the fluorescence responses of a) colloidal NPs from **1** upon exposure to car exhaust gas and b) to a mixture of car exhaust gas and SM vapors at different concentrations. Time‐course curve of the fluorescence responses of c) colloidal NPs from **1** upon exposure to hair spray and d) to a mixture of hair spray and SM vapors at different concentrations.

In conclusion, we developed the two highly photostable and photoluminescent D–A molecules **1** and **2**. Their molecular features, including bulky alkyl chains and the moderate twisting between the D and A moieties, allow for the generation of an HLCT state and the suppression of intermolecular interactions, leading to the high luminescence efficiency of **1** and **2** both in solution and in the solid state. Furthermore, the appropriate A moiety effectively lower the HOMO and LUMO and thus greatly enhance the photostability of **1** and **2**. These D–A molecules with high luminescence efficiency and photostability, exhibit promise in applications as fluorescence sensors for hazardous chemicals. In particular, porous colloidal NPs from **1** have optimized sulfur‐*π* and dipole‐dipole interactions with SM and thus enable the ultrasensitive detection of SM vapor even in complex matrices.

## Experimental Section

##### Fabrication of Aggregates from **1**–**3**

Colloidal NPs from **1** or **2** were self‐assembled by injecting 0.3 mL of the chloroform solution of **1** or **2** (6 mg mL^−1^) into a vial containing 3 mL methanol and aged for 1 day at room temperature. Aggregates from **3** were self‐assembled by injecting 0.3 mL of the dichloromethane solution of **3** (5 mg mL^−1^) into a vial containing 3 mL methanol and aged for 1 day at room temperature. The resulting assemblies that were suspended in solution could be cast onto the substrate and into a quartz tube.

##### Property and Sensing Characterizations

SEM images were obtained on a Hitachi SU8010 field‐emission microscope. SEM samples were prepared by drop‐casting the suspending aggregates in solution onto a silica substrate followed by sputtering Pt on the surface (The sputtered Pt layer was ≈5.4 nm). The fluorescence‐mode optical microscopic images were obtained on an inverted fluorescence microscope (Olympus × 71). UV–vis absorption spectra were obtained on a PerkinElmer Lambda 35. Fluorescence spectra and time‐dependent fluorescence profiles were obtained using an Ocean Optics USB4000 fluorometer with a 385 nm LED lamp as the light source (0.053 mW cm^−2^). The fluorescence quantum yields of the colloidal NPs from **1** and **2** drop‐cast on a PTFE film and the solution (2.5 µM) of **1** and **2** were determined by using a Hamamatsu Absolute PL Quantum Yield spectrometer C11347 coupled with an integrating sphere. Time‐dependent fluorescence profiles of colloidal NPs from **1** and **2** that were drop‐cast one a glass slide as a function of irradiation time were obtained using an Ocean Optics USB4000 fluorometer with a 385 nm LED lamp (0.053 mW cm^−2^) and 365 nm LED lamp (100 mW cm^−2^) as the light source.

The fluorescence sensing of different sulfides and other VOCs was performed using a previously reported home‐built detector.^[^ ^44^, ^45^ ^]^ A jar (40 mL) containing 1 mL of an analyte was sealed for overnight to obtain the saturated vapors. The diluted vapor concentrations of different sulfides and other VOCs were obtained by injecting a small volume of the saturated vapor into a sealed vial (40 mL). Colloidal NPs from **1**, **2**, and aggregates from **3** inside the quartz tube were prepared by casting 10 µL methanol suspension of aggregates into a quartz tube, which were kept to be about 3.3 cm far from the tube air‐inlet. Solvent in the quartz tube was removed with a capillary and then the resulting quartz tube with colloidal NPs inside was dried for 5 min by a blower. Real‐time fluorescence responses were monitored by blowing 5 mL of the analyte vapor at certain concentration into the quartz that would be pumped into the chamber containing the predeposited colloidal NPs by an air pump (150 mL min^−1^). The fluorescence sensing of real SM vapors were performed at the Chemical Defense National Laboratory, where SM vapors at different concentrations were available. The time‐dependent fluorescence intensity and spectra were recorded with an Ocean Optics USB4000 fluorometer using a 385 nm LED lamp as the light source.

##### Theoretical Calculations

Theoretical calculations were performed using the B3LYP/6‐31g (d, p) level of theory in Gaussian 09 package.^[^ ^49^ ^]^

##### Solvatochromic Effect

The influence of the solvent environment on the optical property of a compound can be analyzed using the followed Lippert‐Mataga equation (Equation (1))^[^ ^35^ ^]^ that reveals interactions between solvent and solute.

(1) $$\nu_{a}-\;\nu_{f}=\frac{2{\left(\mu_{e}-\mu_{g}\right)}^{2}}{hca^{3}}\;\Delta f\left(\varepsilon ,n\right)+\mathrm{const}$$

where Δ*f* is the orientational polarizability of a solvent; *µ*_e_ is the excited‐state dipole moment; *µ*_g_ is the ground‐state dipole moment; *ɑ* is the solvent cavity (Onsager) radius. The *ɑ* value of both **1** and **2** was calculated to be 0.85 nm by using Equation (2)^[^ ^21^ ^]^ as shown below:

(2) $$a\;={\left(3M/4N\pi d\right)}^{1/3}$$

The *µ*_g_ values of **1** and **2** were calculated to be 3.88 D and 4.82 D, respectively, by using Gaussian 09 package.^[^ ^49^ ^]^ ∆*f* could be calculated by using Equation (3)^[^ ^21^ ^]^ as shown below:

(3) $$\Delta f\;\left(\varepsilon ,n\right)=\frac{\varepsilon -1}{2\varepsilon +1}\;-\frac{n^{2}-1}{2n^{2}+1}$$

## Conflict of Interest

The authors declare no conflict of interest.

## Supporting information

Supporting Information
