# Supplementary material for: Highly Photostable and Luminescent Donor–Acceptor Molecules for Ultrasensitive Detection of Sulfur Mustard
Source: Adv Sci (Weinh). 2021 Jan 4;8(4):2002615. doi: 10.1002/advs.202002615 (PMC7887598; doi:10.1002/advs.202002615)
Supplement: Supplementary file 1 — Supporting Information [file ADVS-8-2002615-s001.pdf]

WILEY-VCH

© 2020 Wiley-VCH GmbH

ADVANCED SCIENCE
Open Access

Supporting Information

for *Adv. Sci.*, DOI: 10.1002/advs.202002615

Highly Photostable and Luminescent Donor-Acceptor Molecules for Ultrasensitive Detection of Sulfur Mustard

Linfeng Cui,[+] Yanjun Gong,[+] Chuanqin Cheng, Yongxian Guo, Wei Xiong, Hongwei Ji, Lang Jiang,* Jincai Zhao, and Yanke Che**

Supporting Information

Highly Photostable and Luminescent Donor-Acceptor Molecules for Ultrasensitive Detection of Sulfur Mustard

Linfeng Cui,[+] Yanjun Gong,[+] Chuanqin Cheng, Yongxian Guo, Wei Xiong, Hongwei Ji, Lang Jiang,* Jincai Zhao, and Yanke Che**

L. Cui, C. Cheng, Dr. Y. Guo, Dr. W. Xiong, Prof. H. Ji, Prof. L. Jiang, Prof. J. Zhao, Prof. Y. Che
Beijing National Laboratory for Molecular Sciences,
Key Laboratory of Photochemistry,
Institute of Chemistry, Chinese Academy of Sciences, Beijing 100190, China.
University of Chinese Academy of Sciences, Beijing, 100049, China.
E-mail: ykche@iccas.ac.cn, ljiang@iccas.ac.cn, hwji@iccas.ac.cn

Dr. Y. Gong.
Key Laboratory of Colloid and Interface Chemistry Ministry of Education,
School of Chemistry and Chemical Engineering,
Shandong University, Shandong, Jinan 250100, China.

[+] These authors contributed equally to this work.

Contents

Experimental Section

Table S1

Figures S1 to S13

References

Experimental Section

Synthesis of molecule 1

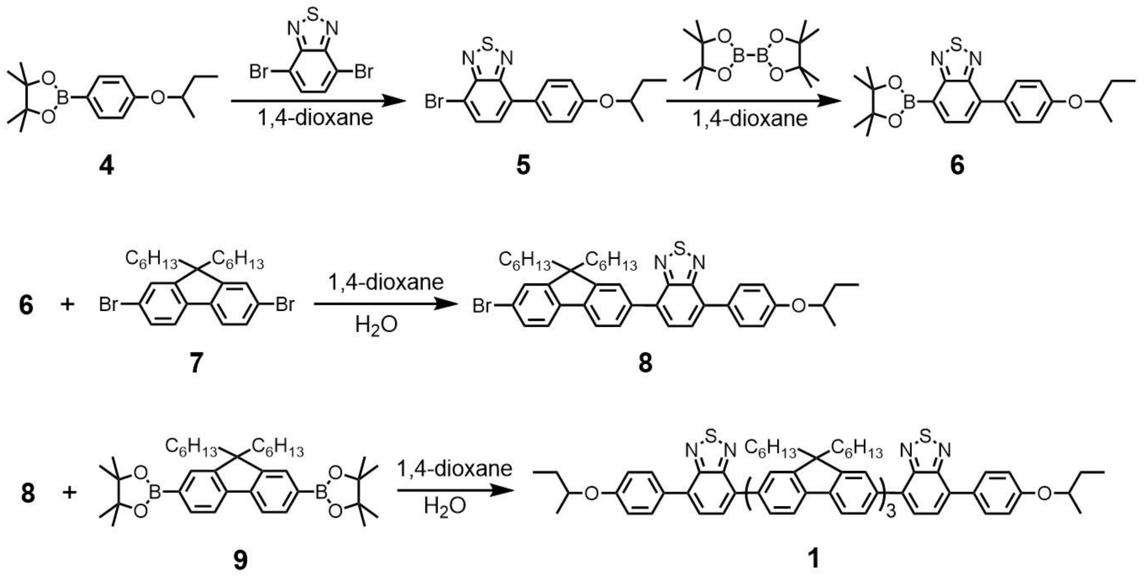

Scheme S1. Synthesis route of molecule **1**.

Molecules **4** and **9** were synthesized by following the previously reported method.[1]

4-bromo-7-(4-(sec-butoxy)phenyl)benzo[c][1,2,5]thiadiazole (5). To a stirred solution of **4** (1.5 g, 5.43 mmol), 4,7-dibromobenzo[c][1,2,5]thiadiazole (1.9 g, 6.5 mmol), and tetrakis(triphenylphosphine)palladium (311 mg, 0.27 mmol) in deoxygenated 1,4-dioxane (40 mL), aqueous solution of potassium carbonate (7 mL, 2.0 M) was added. Then the mixture was heated to 80 °C and stirred overnight under Ar. The solvent was evapourated under vacuum. The residue was poured into water (50 mL) and extracted with ethyl acetate (3 * 40 mL). The combined organic layer was washed with brine (satruated), dried over Na_2SO_4, and concentrated under vacuum. The residue was purified by column chromatography on the silica gel (petroleum: dichloromethane = 10:1 as the eluent) to afford **5** (0.87 g, 2.40 mmol, 44% yield).

Compound **5**: ^{1}H NMR (400 MHz, $CDCl_3$): δ 7.84 (d, *J* = 2.8 Hz, 1 H), 7.82 (d, *J* = 7.6 Hz, 2 H), 7.51 (d, *J* = 7.6 Hz, 1 H), 7.03 (dt, *J* = 8.8 Hz, 2.8 Hz, 2 H), 4.43-4.33 (m, 1 H), 1.82-1.65 (m, 2 H), 1.35 (d, *J* = 6.0 Hz, 3 H), 1.01 (t, *J* = 7.2 Hz, 3 H).

4-(4-(sec-butoxy)phenyl)-7-(4,4,5,5-tetramethyl-1,3,2-dioxaborolan-2-yl)benzo[c][1,2,5]thiadiazole (6). A mixture of **5** (0.87 g, 2.40 mmol), potassium acetate (708 mg, 7.20 mmol), bis(pinacolato)diboron (731 mg, 2.88 mmol), and Pd(dppf)Cl_2 (88 mg, 0.12 mmol) in deoxygenated 1,4-dioxane (20 mL) was heated to 80 °C and stirred overnight under Ar. The solvent was evapourated under vacuum. The residue was poured into water (30 mL) and extracted with ethyl acetate (3 * 20 mL). The combined organic layer was washed with brine (saturated), dried over Na_2SO_4, and concentrated under vacuum. The residue was purified by column chromatography on the silica gel (petroleum: dichloromethane = 5:1 as the eluent) to afford **6** (610 mg, 1.49 mmol, 62% yield).

Compound **6**: ^{1}H NMR (400 MHz, $CDCl_3$): δ 8.23 (d, *J* = 4.0 Hz, 1 H), 7.91 (d, *J* = 8.0 Hz, 2 H), 7.65 (d, *J* = 4.0 Hz, 1 H), 7.03 (7.04, *J* = 8.0 Hz, 2 H), 4.39 (q, *J* = 8.0 Hz, 1 H), 1.83-1.61 (m, 2 H), 1.45 (s, 3 H), 1.34 (t, *J* = 8.0 Hz, 12 H), 1.01 (t, *J* = 8.0 Hz, 3 H).

4-(7-bromo-9,9-dihexyl-9H-fluoren-2-yl)-7-(4-(sec-butoxy)phenyl)benzo[c][1,2,5]thiadiazole (8). To a stirred solution of **6** (368 mg, 0.90 mmol), 2,7-dibromo-9, 9-hexyl-9H-fluorene (**7**) (530 mg, 1.07 mmol), and tetrakis(triphenylphosphine)palladium (51.8 mg, 0.05 mmol) in deoxygenated 1,4-dioxane (20 mL), aqueous solution of potassium carbonate (4 mL, 2.0 M) was added. Then the mixture was heated to 80 °C and stirred overnight under Ar. The solvent was evapourated under vacuum. The residue was poured into water (30 mL) and extracted with ethyl acetate (3 * 30 mL). The combined organic layer was washed with brine (satruated), dried over Na_2SO_4, and concentrated under vacuum. The residue was purified by column chromatography on the silica gel (petroleum: dichloromethane = 2:1 as the eluent) to afford **5** (310 mg, 0.44 mmol, 49% yield).

Compound **8**: ^{1}H NMR (400 MHz, $CDCl_3$): δ 8.01-7.98 (m, 1 H), 7.94-7.92 (m, 3 H), 7.84-7.75 (m, 2 H), 7.81 (d, *J* = 8.0 Hz, 1H), 7.61 (d, *J* = 8.0 Hz, 1H), 7.50-7.47 (m, 2 H), 7.07 (d, *J* = 8.8 Hz, 2 H), 4.48-4.35 (m, 1 H), 2.06-1.96 (m, 4 H), 1.89-1.62 (m, 2 H), 1.38 (d, *J* = 4.0 Hz, 3 H), 1.19-1.05 (m, 12 H), 1.02 (t, *J* = 8.0 Hz, 3 H), 0.79-0.74 (m, 10 H).

7,7'-(9,9,9',9',9'',9''-hexahexyl-9H,9'H,9''H-[2,2',7',2''-terfluorene]-7,7''-diyl)bis(4-(4-sec-butoxy)phenyl)benzo[c][1,2,5]thiadiazole) (1). To a stirred solution of **8** (230 mg, 0.33 mmol), **9** (87.8 mg, 0.15 mmol), and tetrakis(triphenylphosphine)palladium (19 mg, 0.017 mmol) in deoxygenated 1,4-dioxane (20 mL), aqueous solution of potassium carbonate (3 mL, 2.0 M) was added. Then the mixture was heated to 80 °C and stirred overnight under Ar. The solvent was evapourated under vacuum. The residue was poured into water (20 mL) and extracted with ethyl acetate (3 * 20 mL). The combined organic layer was washed with brine (saturated) , dried over Na_2SO_4, and concentrated under vacuum. The residue was purified by column chromatography on silica gel (petroleum: dichloromethane = 1:1 as the eluent) to afford **1** (119 mg, 0.08 mmol, 51% yield). The resulting target compound was confirmed by ^{1}H NMR and MALDI-MS as below.

Molecule **1.** ^{1}H NMR (400 MHz, $CDCl_3$): 8.05 (d, *J* = 8.0 Hz, 2 H), δ 7.96-7.77 (m, 14 H), 7.78 (d, *J* = 8.8 Hz, 2 H), 7.71-7.67 (m, 8 H), 7.08 (d, *J* = 8.8 Hz, 4 H), 4.22 (q, *J* = 6.0 Hz, 2 H), 2.21-2.08 (m, 12 H), 1.91-1.62 (m, 4 H), 1.39 (d, *J* = 4.0 Hz, 6 H), 1.68-1.23 (m, 36 H), 1.05 (t, *J* = 8.0 Hz, 6 H), 0.93-0.82 (m, 12 H), 0.80-0.71 (m, 18 H). MS (MALDI-MS): $[M]^+$ calcd for $C_{107}H_{126}N_4O_2S_2$ 1563.9, found 1563.7.

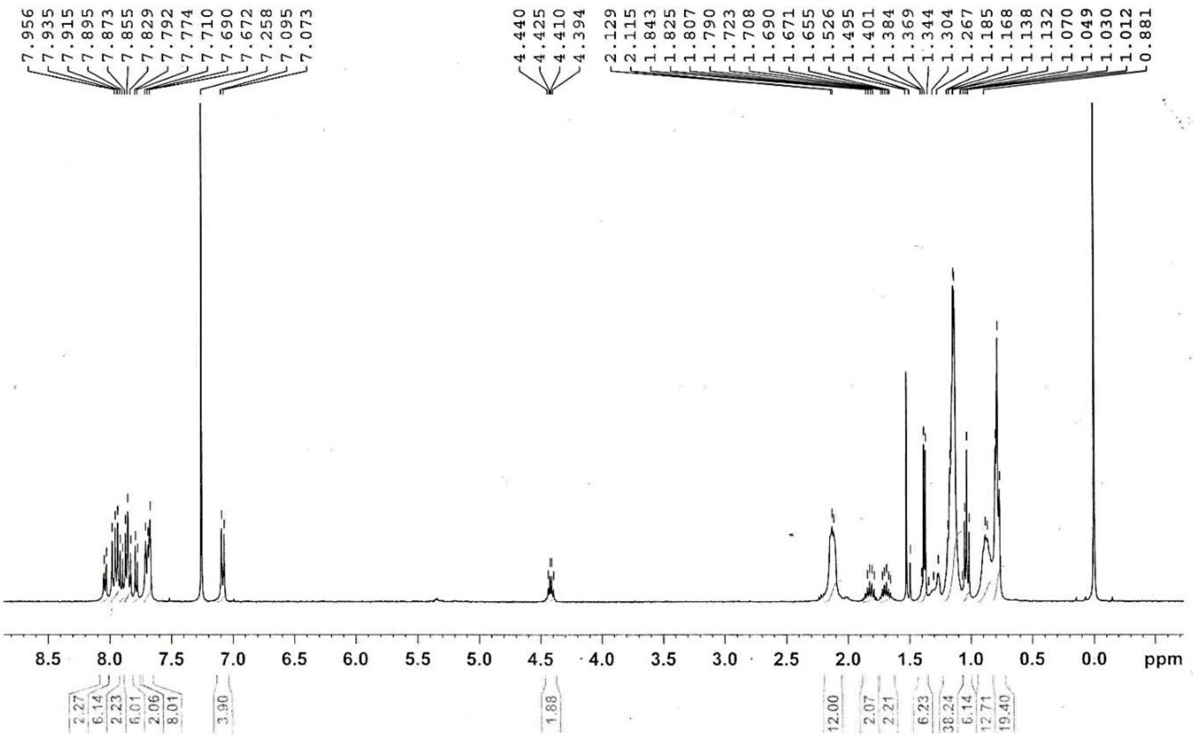
7.956
7.935
7.915
7.895
7.873
7.855
7.829
7.792
7.774
7.710
7.690
7.672
7.258
7.095
7.073
4.440
4.425
4.410
4.394
2.129
2.115
1.843
1.825
1.807
1.790
1.723
1.708
1.690
1.671
1.655
1.526
1.495
1.401
1.384
1.369
1.344
1.304
1.267
1.185
1.168
1.138
1.132
1.070
1.049
1.030
1.012
0.881
8.5 8.0 7.5 7.0 6.5 6.0 5.5 5.0 4.5 4.0 3.5 3.0 2.5 2.0 1.5 1.0 0.5 0.0 ppm
2.27
6.14
2.23
6.01
2.06
8.01
3.90
1.88
12.00
2.07
2.21
6.23
38.24
6.14
12.71
19.40

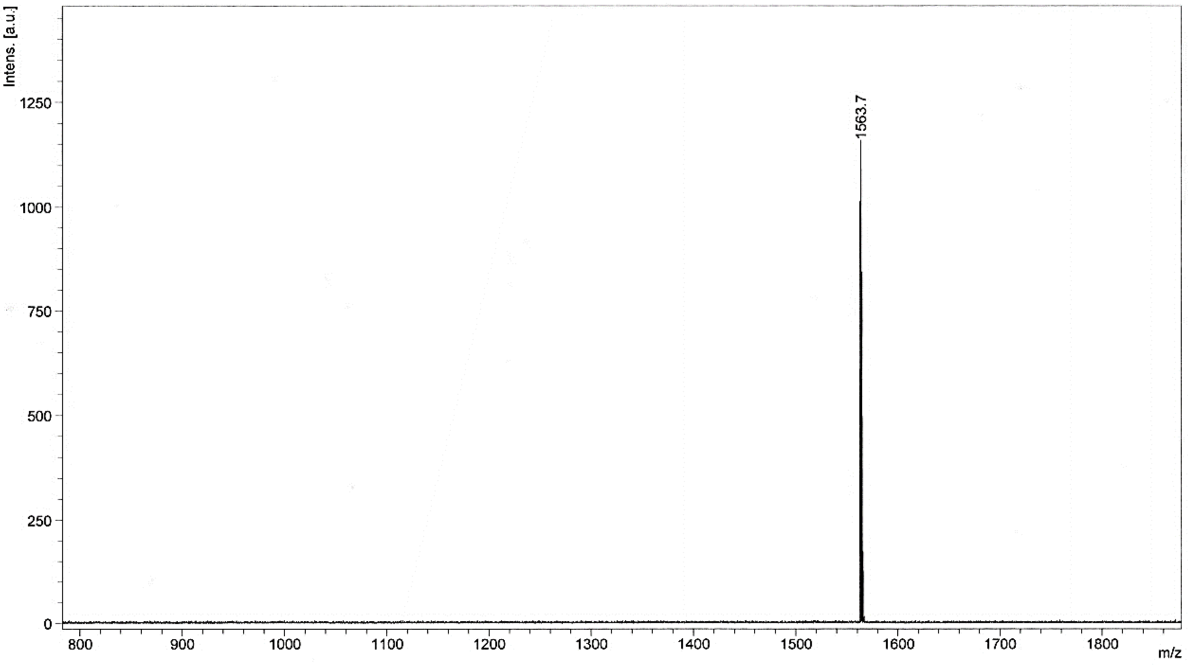
Intens. [a.u.]
1250
1000
750
500
250
0
1563.7
800 900 1000 1100 1200 1300 1400 1500 1600 1700 1800
m/z

Synthesis of molecule 2

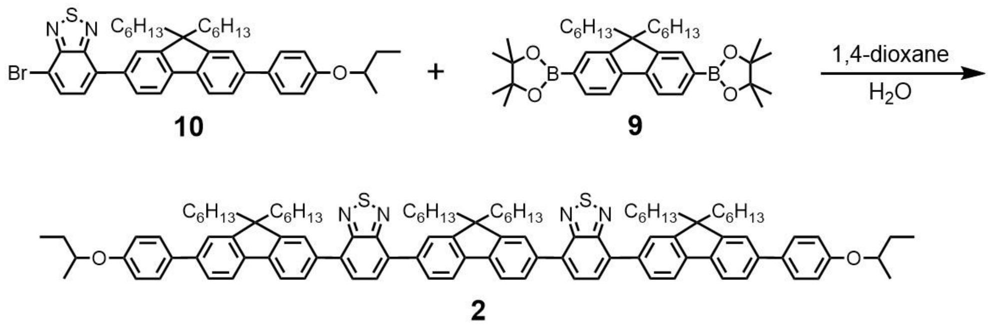

Scheme S2. Synthesis route of molecule **2**.

7,7'-(9,9-dihexyl-9H-fluorene-2,7-diyl)bis(4-(7-(4-(sec-butoxy)phenyl)-9,9-dihexyl-9H-fluoren-2-yl)benzo[c][1,2,5]thiadiazole) (2). To a stirred solution of **10** (300 mg, 0.43 mmol), **9** (121 mg, 0.21 mmol), and tetrakis(triphenylphosphine)palladium (26 mg, 0.022 mmol) in deoxygenated 1,4-dioxane (20 mL), aqueous solution of potassium carbonate (3 mL, 2.0 M) was added. Then the mixture was heated to 80 °C and stirred overnight under Ar. The solvent was evapourated under vacuum. The residue was poured into water (20 mL) and extracted with ethyl acetate (3 * 20 mL). The combined organic layer was washed with brine (saturated) , dried over Na_2SO_4, and concentrated under vacuum. The residue was purified by column chromatography on silica gel (petroleum: dichloromethane = 1:1 as the eluent) to afford **2** (210 mg, 0.13 mmol, 64% yield). The resulting target compound was confirmed by ^{1}H NMR and MALDI-MS as below.

Molecule **2.** ^{1}H NMR (400 MHz, $CDCl_3$): δ 8.08-8.05 (m, 6 H), δ 8.04-7.87 (m, 10 H), 7.80 (d, J = 8.8 Hz, 2 H), 7.62-7.56 (m, 8 H), 7.01 (d, J = 8.8 Hz, 4 H), 4.37 (q, J = 6.0 Hz, 2 H), 2.15-2.03 (m, 12 H), 1.82-1.65 (m, 4 H), 1.35 (d, J = 4.0 Hz, 6 H), 1.25-1.12 (m, 38 H), 1.02 (t, J = 8.0 Hz, 6 H), 0.89-0.72 (m, 28 H). MS (MALDI-MS): $[M]^+$ calcd for $C_{107}H_{126}N_4O_2S_2$ 1563.9, found 1563.9.

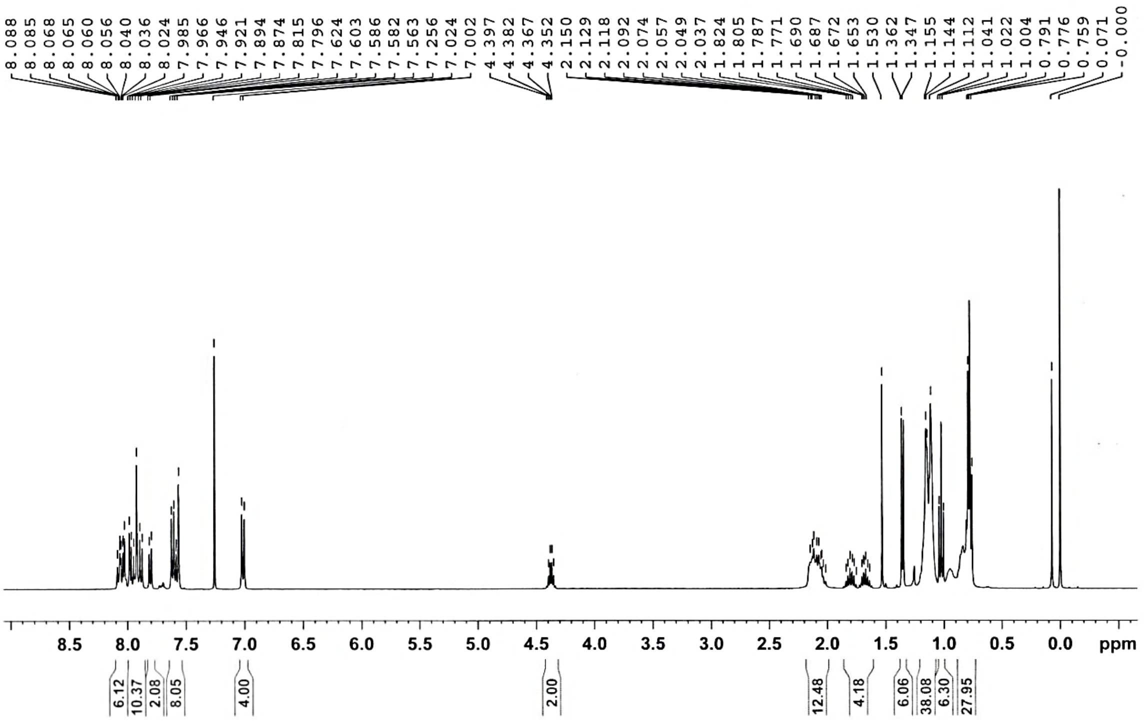
8.5
8.0
7.5
7.0
6.5
6.0
5.5
5.0
4.5
4.0
3.5
3.0
2.5
2.0
1.5
1.0
0.5
0.0
ppm
6.12
10.37
2.08
8.05
4.00
2.00
12.48
4.18
6.06
38.08
6.30
27.95

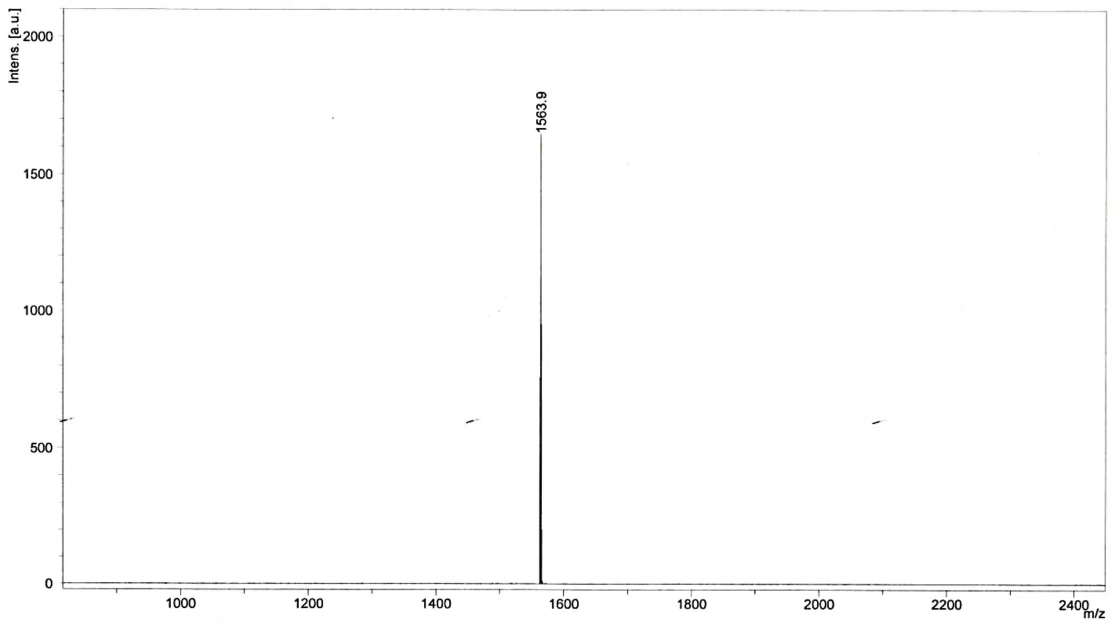
Intens. [a.u.]
1563.9
m/z

Synthesis of molecule 3

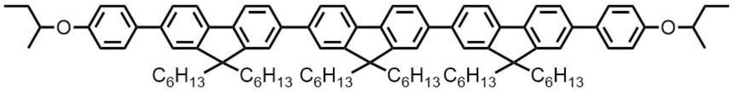

3

Scheme S3. Molecular structure of molecule **3**.

Molecule **3** was synthesized by following the previously reported method.[1]

Molecule **3**. ^{1}H NMR (400 MHz, $CDCl_3$): δ 7.76 (m, 6 H), 7.68-7.54 (m, 16 H), 7.02 (d, *J* = 8.4 Hz, 4 H), 4.38 (m, 2 H), 2.12-2.06 (m, 12 H), 1.85-1.62 (m, 4 H), 1.36-1.09 (m, 48 H), 1.02 (m, 30 H). MS (MALDI-MS): $[M]^+$ calcd for $C_{95}H_{122}O_2$ 1294.9, found 1295.0.

Table S1

Table S1. Photophysical data of **1** and **2** in different solvents (2.5 µM).

Solvent	M	Δf[2]	λ_{abs} a) [nm]	λ_{em} b) [nm]	Stokes' shift (ν_a–ν_f) [cm^{-1}]	Φ_F c) [%]
Cyclohexane	**1**	0	418	522	4766	97
	**2**	0	437	523	3763	100
Toluene	**1**	0.014	419	532	5069	100
	**2**	0.014	440	534	4001	100
Butyl ether	**1**	0.096	414	527	5179	99
	**2**	0.096	439	531	3947	100
Isopropyl ether	**1**	0.145	416	527	5063	98
	**2**	0.145	438	532	4034	100
Chloroform	**1**	0.149	420	549	5595	100
	**2**	0.149	441	548	4428	100
Ether	**1**	0.167	417	531	5148	100
	**2**	0.167	436	534	4209	100
Ethyl acetate	**1**	0.200	411	539	5778	100
	**2**	0.200	436	541	4451	100
Tetrahydrofuran	**1**	0.210	419	543	5450	100
	**2**	0.210	439	546	4464	100
Dichloromethane	**1**	0.218	415	552	5980	100
	**2**	0.218	437	554	4833	100
N,N-Dimethylformamid	**1**	0.276	419	567	6230	98
	**2**	0.276	441	569	5101	97
Acetone	**1**	0.284	412	555	6254	100
	**2**	0.284	436	557	4982	100

a)Absorption maximum; b)Emission maximum; c)Fluorescence quantum yield.

Figure S1 to S13

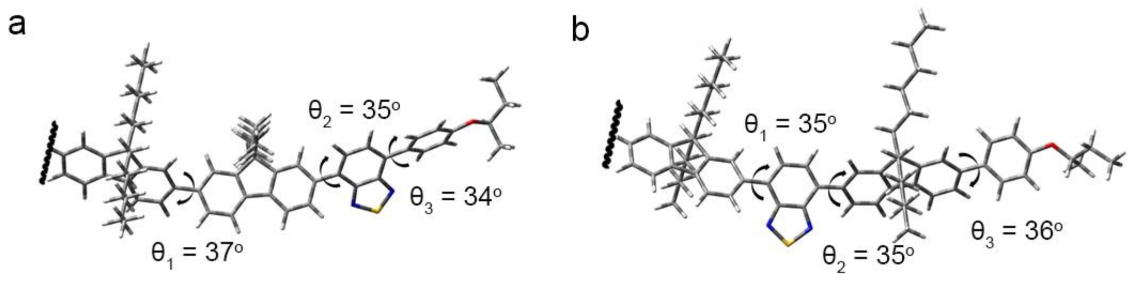

Figure S1. Molecular configuration of **1** (a) and **2** (b) optimized by Gaussian 09 package.[3]

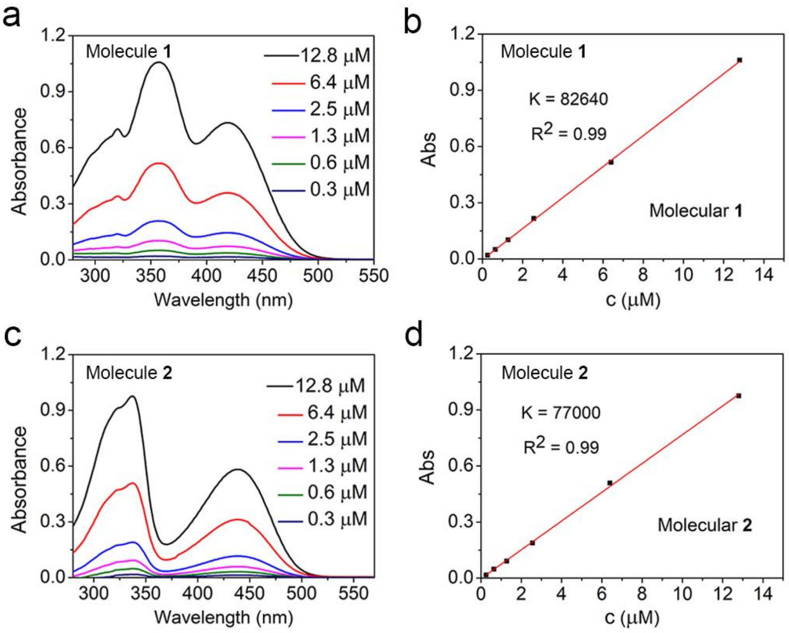

Figure S2. a) Absorption spectra of **1** in chloroform at different concentrations. b) Linear correlation of the maximum absorbance and concentration of **1**. c) Absorption spectra of **2** in chloroform at different concentrations. d) Linear correlation of the maximum absorbance and concentration of **2**.

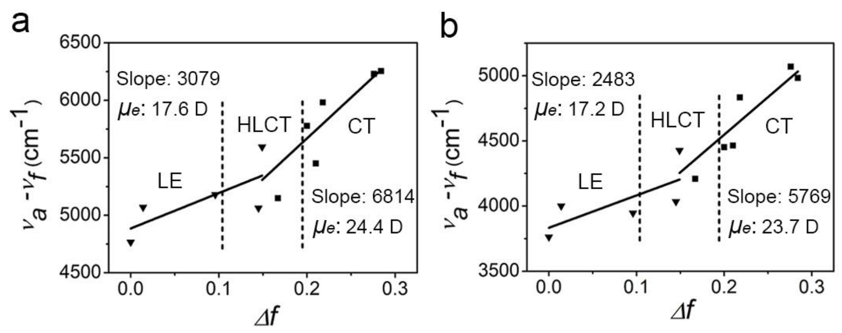

Figure S3. Linear correlation of the orientation polarity (Δf) [2] of solvents with the Stokes' shift (ν_a–ν_f) of **1** (a) and **2** (b).

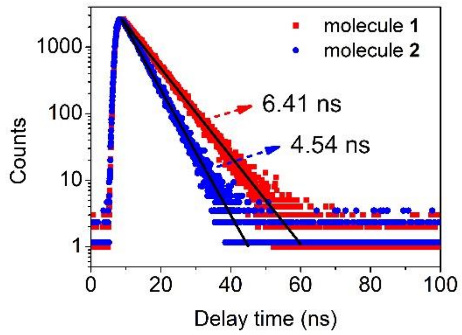

Figure S4. Flourescence lifetime measurements of **1** (a) and **2** (b) in chloroform (2.5 μM).

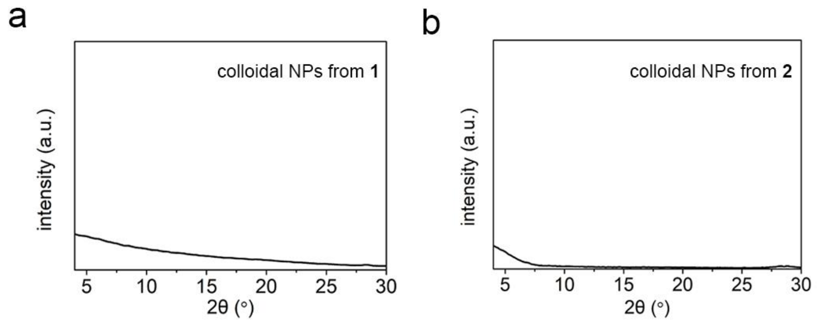

Figure S5. XRD results of colloidal NPs from **1** (a) and **2** (b).

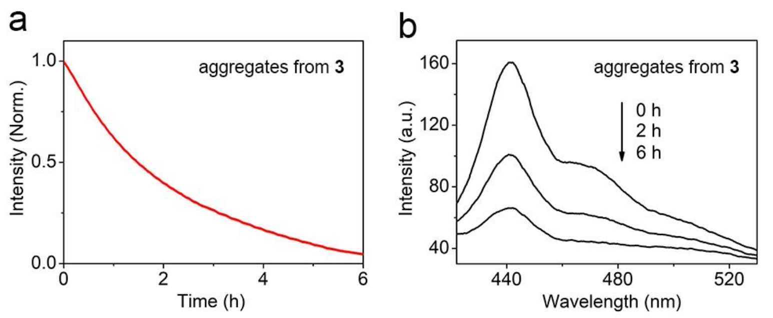

Figure S6. a) Fluorescence intensity of aggregates from **3** monitored in the range of 425-465 nm as a function of irradiation time (385 nm, 0.053 mW cm^{-2}). b) Time-dependent fluorescence spectra of aggregates from **3.**

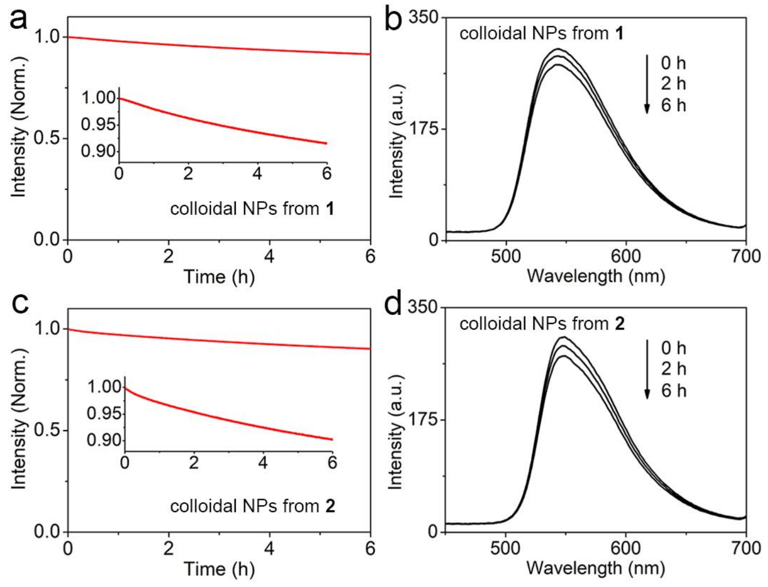

Figure S7. a, c) Fluorescence intensity of colloidal NPs from **1** (a) and **2** (c) monitored in the range of 520-560 nm as a function of irradiation time (365 nm, 100 mW cm^{-2}). The insets shows zoomed-in plots. b, d) Time-dependent fluorescence spectra of colloidal NPs from **1** (b) and **2** (d) as a function of irradiation time (365 nm, 100 mW cm^{-2}).

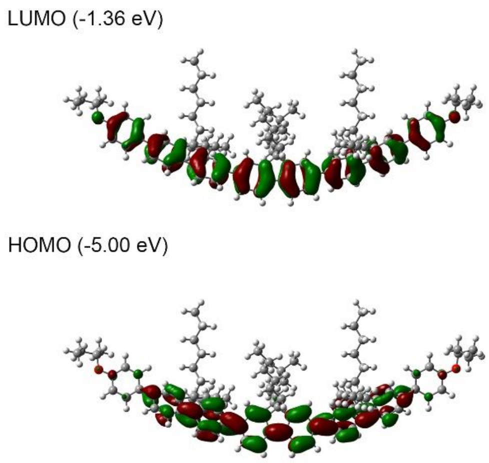

Figure S8. Frontier molecular orbitals of molecule **3** calculated by Gaussian 09 package.[3]

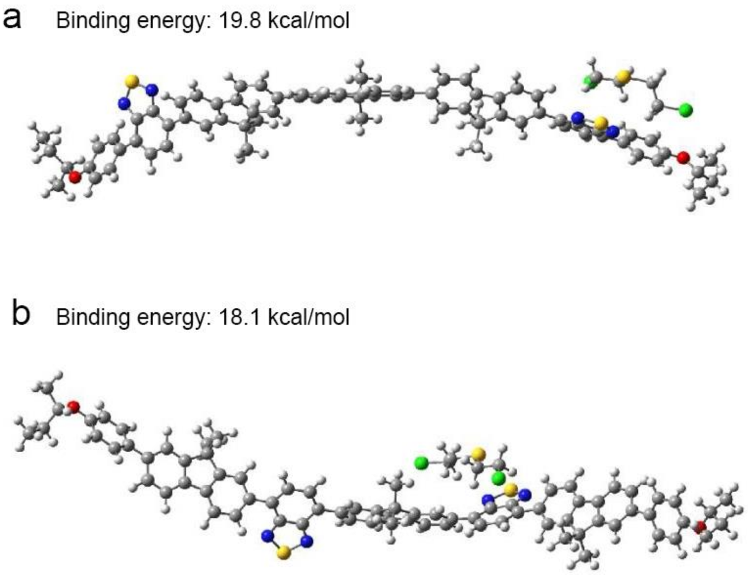

Figure S9. a, b) The binding energy between molecule **1** (a) or molecule **2** (b) and SM calculated at the B3LYP/6-31G level.[3]

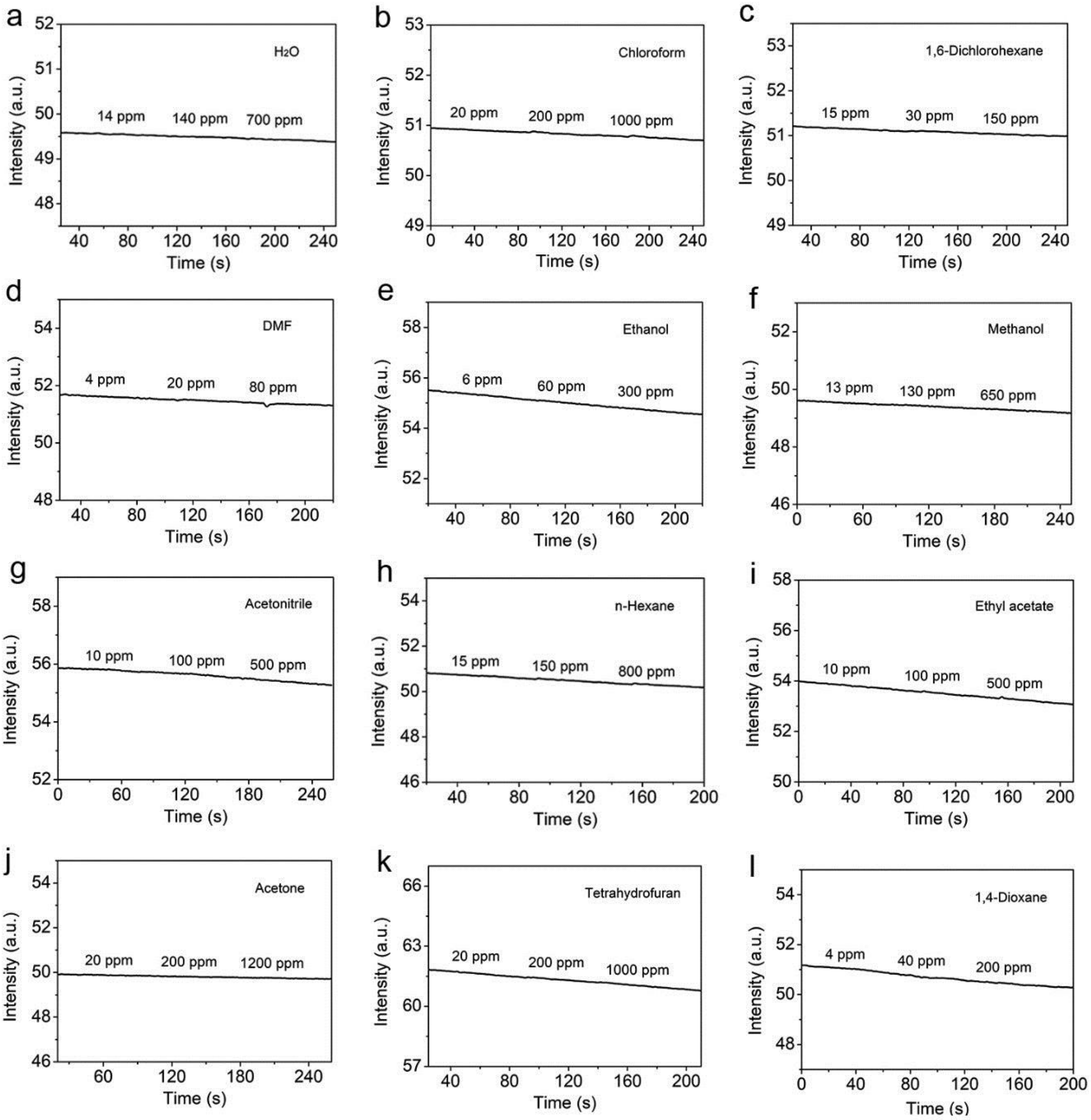

Figure S10. a-l) Time-course curves of the fluorescence response of colloidal NPs from **1** upon exposure to various VOCs at different concentrations.

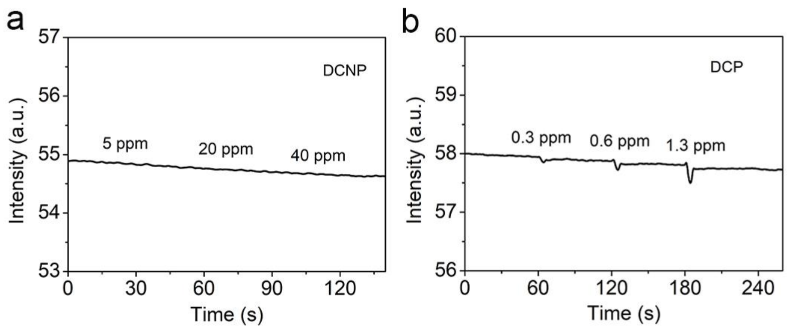

Figure S11. a) Time-course curve of the fluorescence response of colloidal NPs from **1** upon exposure to DCNP vapors at different concentrations. b) Time-course curve of the fluorescence response of colloidal NPs from **1** upon exposure to DCP vapors at different concentrations.

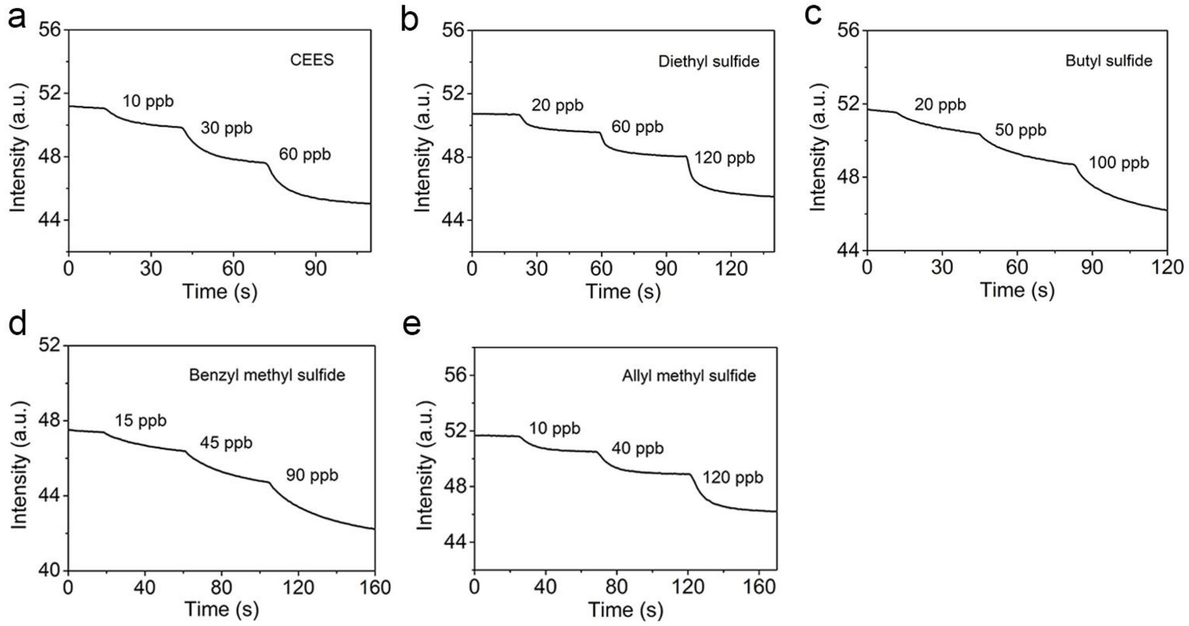

Figure S12. a-e) Time-course curves of the fluorescence response of colloidal NPs from **1** upon exposure to CEES, dimethyl sulfide, butyl sulfide, benzyl methyl sulfide, and allyl methyl sulfide vapors at different concentrations.

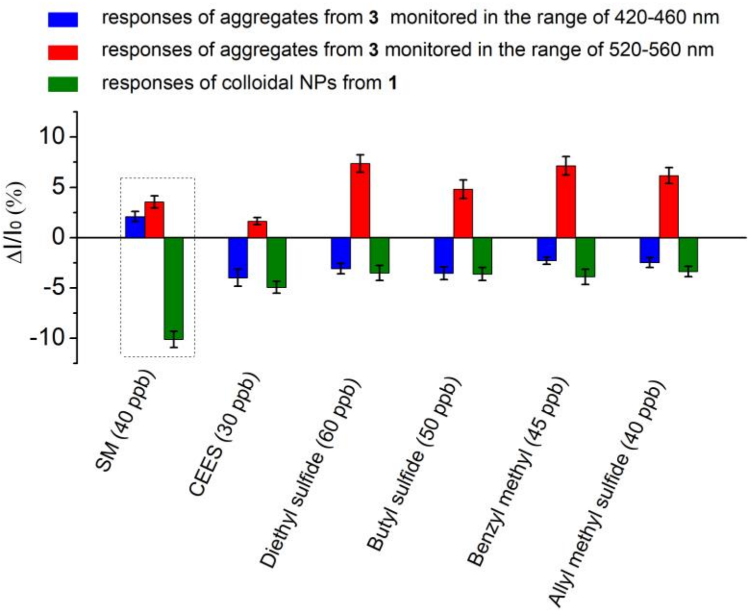

Figure S13. Fluorescence responses of aggregates from **3** and colloidal NPs from **1** to SM and different sulfides. $\Delta I/I_0$ represents the ratio of the changed fluorescence intensity to the original intensity. Error bars represent the standard deviation of five measurements.

References

[1] C. Qiu, X. Liu, C. Cheng, Y. Gong, W. Xiong, Y. Guo, C. Wang, J. Zhao, Y. Che, *Anal. Chem.* **2019**, *91*, 6408.

[2] W. Li, D. Liu, F. Shen, D. Ma, Z. Wang, T. Feng, Y. Xu, B. Yang, Y. Ma, *Adv. Funct. Mater.* **2012**, *22*, 2797.

[3] M. J. T., G. W. Frisch, H. B. Schlegel, G. E. Scuseria, M. A. Robb, J. R. Cheeseman, G. Scalmani, V. Barone, B. Mennucci, G. A. Petersson, H. Nakatsuji, M. Caricato, X. Li, H. P. Hratchian, A. F. Izmaylov, J. Bloino, G. Zheng, J. L. Sonnenberg, M. Hada, M. Ehara, K. Toyota, R. Fukuda, J. Hasegawa, M. Ishida, T. Nakajima, Y. Honda, O. Kitao, H. Nakai, T. Vre.ven, J. A., Jr. Montgomery, J. E. Peralta, F. Ogliaro, M. Bearpark, J. J. Heyd, E. Brothers,

K. N. Kudin, V. N. Staroverov, T. Keith, R. Kobayashi, A. J. Norm, K. Raghavachari, A. Rendell, J. C. Burant, S. S. Iyengar, J. Tomasi, M. Cossi, N. Rega, J. M. Millam, M. Klene, J. E. Knox, J. B. Cross, V. Bakken, C. Adamo, J. Jaramillo, R. Gomperts, R. E. Stratmann, O. Yazyev, A. J. Austin, R. Cammi, C. Pomelli, J. W. Ochterski, R. L. Martin, K. Morokuma, V. G. Zakrzewski, G. A. Voth, P. Salvador, J. J. Dannenberg, S. Dapprich, A. D. Daniels, O. Farkas, J. B. Foresman, J. V. Ortiz, J. Cioslowski, D. J. Fox, *Gaussian 09, Revision B.01, Gaussian, Inc.: Wallingford CT,* **2010**.
